# 292. Phosphorodiamidate Morpholino Oligomers Targeting *acpP* Reduce the Biofilm Burden in *Burkholderia cepacia* Complex

**DOI:** 10.1093/ofid/ofad500.364

**Published:** 2023-11-27

**Authors:** Antonio R Mendez, Christine A Pybus, David E Greenberg

**Affiliations:** University of Texas Southwestern Medical Center, Dallas, Texas; UT Southwestern Medical Center, Dallas, TX; UT Southwestern, Dallas, Texas

## Abstract

**Background:**

The *Burkholderia cepacia* complex (Bcc) is composed of highly related opportunistic species that establish pulmonary infections in immunodeficient hosts, such as those with cystic fibrosis and chronic granulomatous disease. Due to their innate antibiotic-resistant phenotypes and capacity to form biofilm, Bcc infections are difficult to eradicate with traditional antibiotics. Peptide-conjugated phosphorodiamidate morpholino oligomers (PPMOs) offer an alternative therapeutic approach. These nucleotide analogues penetrate the outer membrane, bind specific mRNA, and inhibit translation. Due to high affinity base-pairing dynamics, these molecules can be rapidly modified and deployed to precisely inactivate innumerable bacterial gene targets. Previously, we found PPMOs targeting an acyl carrier protein (AcpP) are bactericidal against planktonic Bcc. It remains unknown whether PPMOs could modulate biofilm formation in these pathogens.

Proposed Mechanism of Action of PPMOs

**Figure:**
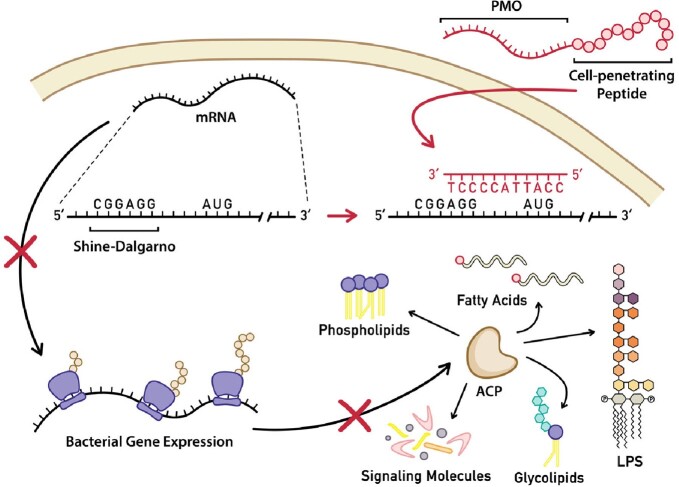

Inside a bacterial cell, peptide-conjugated phosphorodiamidate morpholino oligomers (PPMOs) targeting the acpP gene bind to mRNA near the Shine-Dalgarno sequence and AUG start site, sterically inhibiting translation. This inhibition disrupts the expression and essential functions of acyl carrier protein (ACP).

**Methods:**

Minimum biofilm eradication concentration assays were used to test two AcpP PPMOs on 24 h biofilms formed on pegs. Viable cells were enumerated. This activity was further evaluated by microscopy and 7-day biofilm kinetic studies. Fluorescein-labeled AcpP PPMO added to *B. cenocepacia* K56-2 DsRed was imaged over time to evaluate colocalization. PPMO toxicity was assessed over 24 h in A549 cells by LDH release.

**Results:**

Treatment (10-40 μM) with AcpP PPMOs demonstrated more than a 3-log reduction (*p*< 0.0001) in biofilm burden across five clinical isolates of Bcc tested and *B. thailandensis* E264. PPMO bactericidal activity was visualized with confocal and scanning electron microscopy. Fluorescently labeled AcpP PPMO associated with the membrane of *B. cenocepacia* K56-2 DsRed in a temporal fashion. Additionally, AcpP PPMOs exhibited low toxicity in human pneumocytes.

**Conclusion:**

PPMOs are active and bactericidal in established Bcc biofilms; thus, the biofilm setting is not a deterrent against PPMO delivery. This is further supported by the observations that AcpP PPMOs and Bcc cells colocalize in biofilm, resulting in membrane destruction and biomass reduction. Together, these data provide evidence that PPMOs are active in the biofilm setting of Bcc and could be a promising therapeutic strategy for these infections.

**Disclosures:**

**David E. Greenberg, MD**, University of Texas Southwestern Medical Center: Dr. Greenberg has numerous patents on PPMOs

